# Generation of Recombinant Rabies Virus CVS-11 Expressing eGFP Applied to the Rapid Virus Neutralization Test

**DOI:** 10.3390/v6041578

**Published:** 2014-04-04

**Authors:** Xianghong Xue, Xuexing Zheng, Hongru Liang, Na Feng, Yongkun Zhao, Yuwei Gao, Hualei Wang, Songtao Yang, Xianzhu Xia

**Affiliations:** 1 Institute of Military Veterinary Medicine, Academy of Military Medical Science, Changchun 130122, China; E-Mails: red99.smile@163.com (X.X.); zhengxx2513@126.com (X.Z.); hrliang13@126.com (H.L.); fengna0308@126.com (N.F.); zhaoyongkun1976@126.com (Y.Z.); gaoyuwei@gmail.com (Y.G.); wangh25@hotmail.com (H.W.); 2 College of Veterinary Medicine, Jilin University, Changchun 130062, China; 3 Biotechnology Research Institute, Chinese Academy of Agricultural Sciences, Beijing 100081, China

**Keywords:** rabies virus, eGFP, virus-neutralizing antibody (VNA), FAVN

## Abstract

The determination of levels of rabies virus-neutralizing antibody (VNA) provides the foundation for the quantitative evaluation of immunity effects. The traditional fluorescent antibody virus neutralization test (FAVN) using a challenge virus standard (CVS)-11 strain as a detection antigen and staining infected cells with a fluorescein isothiocyanate (FITC)-labeled monoclonal antibody, is expensive and high-quality reagents are often difficult to obtain in developing countries. Indeed, it is essential to establish a rapid, economical, and specific rabies virus neutralization test (VNT). Here, we describe a recombinant virus rCVS-11-eGFP strain that stably expresses enhanced green fluorescent protein (eGFP) based on a reverse genetic system of the CVS-11 strain. Compared to the rCVS-11 strain, the rCVS-11-eGFP strain showed a similar growth property with passaging stability *in vitro* and pathogenicity *in vivo*. The rCVS-11-eGFP strain was utilized as a detection antigen to determine the levels of rabies VNAs in 23 human and 29 canine sera; this technique was termed the FAVN-eGFP method. The good reproducibility of FAVN-eGFP was tested with partial serum samples. Neutralization titers obtained from FAVN and FAVN-eGFP were not significantly different. The FAVN-eGFP method allows rapid economical, specific, and high-throughput assessment for the titration of rabies VNAs.

## 1. Introduction

Rabies is a fatal zoonotic infectious disease caused by the rabies virus (RABV) that affects numerous warm-blooded mammals worldwide [1]. Rabies is almost 100% fatal once clinical signs are present in infected humans or animals [2]. Globally, an estimated 55,000 human deaths are attributed to RABV annually, including 3000 cases in China [3,4]. Dogs and cats are the major reservoir of human rabies in most parts of the developing world [5], and the vast majority of human rabies victims become infected by dog bites [6,7]. Studies by the World Health Organization (WHO) have shown that 70% immunization of the dog population can efficiently block RABV transmission [4,8], and that virus-neutralizing antibody plays an important role in protecting against RABV [9,10]. However, quantification of the VNA levels against RABV is essential for the timely monitoring of immunization coverage in dog and cat populations and to evaluate the immunity effectiveness of RABV vaccines. According to the WHO, vaccinated animals are sufficiently protected from RABV when the levels of rabies VNA equal or exceed 0.5 IU/mL [11]. The principle of the detection and quantification of VNA titers is that the cells or animals are infected with viruses not inhibited by antibodies *in vitro* or *in vivo*. FAVN and the rapid fluorescent focus inhibition test (RFFIT) approved by the Office International Des Epizooties (OIE) and WHO, respectively, have been widely used to measure levels of VNAs [12,13].

The CVS-11 strain was widely used in determination of virus-neutralizing antibody and the eGFP has been widely used as a tracer protein to assess virus rescue efficiency [13,14,15,16] and to study the characteristics and localization of viruses [17,18]. In this study, we generated a recombinant RABV rCVS-11-eGFP strain harboring an eGFP gene in the genome. The rCVS-11-eGFP strain was used as a challenge virus to detect the levels of the virus-neutralizing antibodies. This modified FAVN method is termed FAVN-eGFP. Finally, the traditional FAVN and novel FAVN-eGFP methods were compared.

## 2. Experimental Section

### 2.1. Viruses and Cells

The RABV challenge virus standard (CVS)-11 strain was kindly provided by the institute for Viral Disease Prevention and Control (Beijing, China). Neuroblastoma (NA) cells of A/J mouse origin were grown in Dulbecco’s modified Eagle’s MEM (DMEM) supplemented with 10% fetal bovine serum (FBS). Baby hamster kidney (BHK)-21 cells were grown in Dulbecco’s modified Eagle’s MEM (DMEM) supplemented with 10% FBS. The wtCVS-11, rCVS-11 and rCVS-11-eGFP strain were propagated in BHK-21 cells cultured in DMEM with 2% FBS for virus stock preparation. The viruses were stored at −70 °C before use.

### 2.2. Serum Samples

Nineteen serum samples were obtained from dogs receiving vaccination with Rabisin (Pitman-Moore strain, Paris, France). Twenty-three serum samples were obtained from volunteers receiving rabies preexposure immunization with rabies vaccines (PV2026 strain, Shenyang, China). These serum samples were used with the permission of the volunteers under Good Clinical Practice (GCP) guidelines. Ten negative dog serum samples were obtained from non-vaccinated dogs. All serum samples were stored at −20 °C and were inactivated at 56 °C for 30 min prior to testing.

### 2.3. Construction of the Recombinant rCVS-11-eGFP Strain

**Figure 1:**
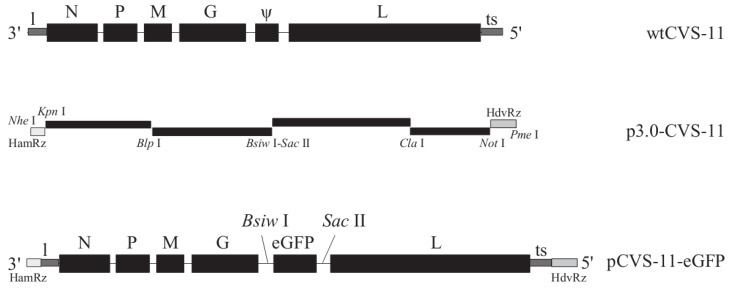
Schematic construction of the full-length cDNA recombinant plasmid. The F1–F4 fragments covering the entire RABV genome of wtCVS-11 strain were amplified and inserted into pcDNA3.1. An eGFP gene from the eukaryotic expression vector pIRES2-eGFP was then cloned at the *Bsiw* I/*Sac* II sites to generate recombinant pCVS-11-eGFP.

Construction of the RABV CVS-11 strain full-length cDNA recombinant vector (p3.0-CVS-11) was described elsewhere [19]. The open reading frame (ORF) of the eGFP gene was amplified with primers 5'-CGTACGCACAACCATGGTGAGCAAG-3' (the *Bsiw* I site is underlined) and 5'-CCGCGGTTACTTGTACAGCTCGTCCATG-3' (the *Sac* II site is underlined) for pIRES2-EGFP (Invitrogen, Carlsbad, CA, USA) and inserted between the *Bsiw* I and *Sac* II sites in p3.0-CVS-11. The resulting plasmid was designated pCVS-11-eGFP (Figure 1). All primer sequences used in this study are available from the corresponding author upon request.

### 2.4. Recovery of the Recombinant rCVS-11-eGFP Strain from Cloned cDNA

The rCVS-11-eGFP strain generated from the full-length plasmid pCVS-11-eGFP was rescued as described [20]. Briefly, 2 × 10^5^ NA cells per well were grown overnight to 60%–80% confluence in six-well plates (Corning, Steuben County, NY, USA) in DMEM supplemented with 10% FBS. The cells were transfected with 2 µg of pCVS-11-eGFP with 0.5 µg pcDNA3.1-N, 0.25 µg pcDNA3.1-P, 0.15 µg pcDNA3.1-G, and 0.1 µg pcDNA3.1-L using SuperFect Transfection Reagent (Qiagen, Hilden, Germany). After 3 h, the transfection medium was replaced with fresh DMEM plus 10% FBS. The supernatants were transferred onto new NA cells five days later and incubated for another three days for virus propagation. A direct fluorescence assay (DFA) was performed for the detection of viral N protein from rescued RABV using a FITC-labeled RABV N protein-specific monoclonal antibody (Centocor, Malvern, PA, USA). The green fluorescence in the cells infected with the rCVS-11-eGFP strain was measured under a fluorescence microscope at two days postinfection (p.i.). The rescued virus rCVS-11-eGFP strain was inoculated in BHK-21 cells for virus stock preparation and for further experiments.

### 2.5. Confirmation of the rCVS-11-eGFP Strain

To determine whether recombinant rCVS-11-eGFP was derived from pCVS-11-eGFP, RT-PCR was performed using primers based on the wtCVS-11 genome: sense, DF-4805 (5'-ACAGGGGGGAATGTGTCAGTC-3'), and anti-sense DR-5587, (5'-TGTTCCCTGTCTTCAACCATTC-3'). The supernatant from BHK-21 cells infected with rCVS-11-eGFP at a multiplicity of infection (MOI) of 0.1 was harvested at five days p.i. The virus in the supernatant was concentrated by ultracentrifugation (120,000× *g*, 120 min) and suspended in phosphate-buffered saline (PBS). A 4% solution of phosphotungstic acid (pH 6.8) was added to the virus suspension for negative staining; the mixtures were transferred to carbon-coated grids, and the specimens were photographed with an electron microscope H8100 (Hitachi Ltd., Hitachi, Japan).

### 2.6. Virus Multistep Growth Assay

Monolayers of NA or BHK-21 cell grown in six-well plates were infected with rCVS-11 or rCVS-11-eGFP at an MOI of 0.1. After inoculation for 1 h at 37 °C, the inocula were removed, and the cells were washed twice with PBS (pH 7.4). The NA or BHK-21 cells were supplied with DMEM containing 2% FBS and incubated in a 5% CO_2_ incubator at 37 °C. A 100 μL aliquot of the supernatant was collected from the NA or BHK-21 cells infected with rCVS-11-eGFP at 24 h, 48 h, 72 h, 96 h, and 120 h p.i. for virus titration. The titers at each time point were obtained in triplicate.

### 2.7. Pathogenicity of rCVS-11-eGFP in Mice

Ten three-day-old and ten six-week-old female Balb/C mice (Changchun Institute of Biological Products, Changchun, China) were intracranially (i.c.) inoculated with 10^4^ TCID_50_ of the recombinant rCVS-11-G strain and observed for 15 days. The original strain wtCVS-11 was used parallel.

### 2.8. Fluorescent Antibody Virus Neutralization Tests (FAVN)

The FAVN test, a modified RFFIT method, and the titration of rabies VNA in serum samples and WHO reference positive and negative sera were performed using 96-well plates and CVS-11 as challenge virus, as previously described [21]. Briefly, three-fold dilutions (0.05 mL) of the examined sera (1:3, 1:9, 1:27, 1:81, 1:243, and 1:19683), including the WHO reference positive and negative sera, were prepared directly in plates and repeated four times for each sample. Aliquots (0.05 mL) of the virus dilutions containing approximately 100 TCID_50_ wtCVS-11 viruses were added to the serum dilution well. After incubation for 60–90 min, 0.05 mL of cell suspension containing 5 × 10^4^ BHK-21 cells was added to serum-virus mixture. After incubation for 48 h, the cells were fixed with 80% acetone. After air-drying, the cells were stained with FITC- conjugated anti-N protein monoclonal antibody (1:200) and Evans blue (1:500) for 60 min. The entire surface of each well was evaluated, and the results were obtained using the “all or nothing” method. The results were calculated according to Spearman and Kärber [22]. The values of VNA titers were obtained by comparing the 50% effective dose (ED_50_) of the examined serum with ED_50_ of the WHO reference positive serum diluted to 0.5 IU/mL. Fifty-two serum samples from dogs and human were examined with the FAVN test by the OIE Reference Laboratory for rabies diagnosis.

### 2.9. The FAVN-eGFP Method

Compared to conventional FAVN using wtCVS-11 as the challenge virus, the novel FAVN-eGFP method uses the rCVS-11-eGFP strain as the challenge virus. The novel FAVN-eGFP method was as follows. A 0.05 mL aliquot of three-fold dilutions of the WHO reference positive and negative sera and the examined serum samples were added to 96-well plates, with each serum sample repeated four times. Virus dilutions (0.05 mL) containing approximately 100 TCID_50_ of the rCVS-11-eGFP strain were added to the serum dilution in the wells. After incubation at 37 °C for 60 min in a 5% CO_2_ incubator, 0.05 mL of the cell suspension containing 5 × 10^4^ BHK-21 cells was added to each well. After incubation at 37 °C for 60 h in a 5% CO_2_ incubator, the cells were directly observed under a fluorescence microscope, and the results were recorded using the “all or nothing” method. The entire surface of each well was evaluated. The plates containing the cells control test, titration of the rCVS-11-eGFP strain, and titration of the reference sera were evaluated first, followed by the plates with the examined serum samples. The results were calculated according to Spearman and Kärber [22]. The values of VNA titers were obtained by comparing the ED_50_ values of the examined sera with the ED_50_ values of the WHO reference positive serum diluted to 0.5 IU/mL.

Portion of serum samples were tested with our FAVN-eGFP at least twice. Statistical analyses of the repeated results were performed for significant difference. 

### 2.10. Data Analyses

All data were analyzed by paired two-tailed Student’s t-test, one-way ANOVA in SPSS 13.0 software [23]. For all tests, the following notation is used to indicate no significance between groups: *p* > 0.05.

## 3. Results

### 3.1. Recovery of the rCVS-11-eGFP Strain

**Figure 2:**
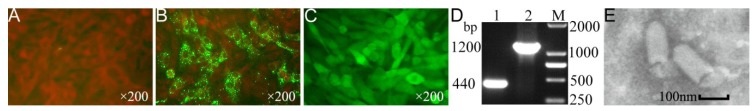
Identification of the recombinant RABV rCVS-11-eGFP strain. (**A**) Negative control. BHK-21 cells were fixed with 80% cold acetone and stained with an FITC-labeled anti-N protein monoclonal antibody (1:200) and Evans blue (1:500, diluted with PBS, pH 7.4) for 1 h; (**B**) NA cells transfected with the pCVS-11-eGFP and pCVS-N, -P, -G, and -L plasmids were incubated for five days, and the supernatants were transferred onto newly prepared BHK-21 cells for another three days. These BHK-21 cells were fixed with 80% cold acetone and stained with the FITC-labeled anti-N protein monoclonal antibody (1:200) and Evans blue (1:500, diluted with PBS, pH 7.4) for 1 h at five days p.i.; (**C**) eGFP was expressed in BHK-21 cells infected with rCVS-11-eGFP at an MOI of 1 p.i. for 60 h; (**D**) Confirmation of the recovery of the rCVS-11-eGFP strain from the cloned cDNA by RT-PCR. Lane 1, RT-PCR products of the recovered rCVS-11 strain were electrophoresed through a 1% agarose gel (length 440 bp); Lane 2, RT-PCR products of the recovered rCVS-11-eGFP strain were electrophoresed through a 1% agarose gel (length 1200 bp); (**E**) The rCVS-11-eGFP strain was grown in BHK-21 cells, and the viral supernatant was pelleted by ultracentrifugation and suspended in PBS (pH 7.4). The rCVS-11-eGFP virions were negatively stained and observed by electron microscopy.

To verify the rescued RABV rCVS-11-eGFP strain replication and expression of eGFP as an RABV structural protein, BHK-21 cells infected with rCVS-11-eGFP were immunostained with FITC-conjugated anti-N protein monoclonal antibody at three days p.i. (Figure 2B); negative BHK-21 cells are shown as Figure 2A. The BHK-21 and 293T cells infected with the rCVS-11-eGFP strain at an MOI of 1 were directly observed for green fluorescence under a fluorescence microscope at three days p.i. (Figure 2C). To confirm that the rCVS-11-eGFP strain was derived from the full-length plasmid pCVS-11-eGFP, BHK-21 cells infected with the rCVS-11-eGFP strain were used to perform RT-PCR of the region between the G and L genes with primers DF-4805 and DR-5587. Amplified fragments of the rCVS-11 or rCVS-11-eGFP strain with the expected sizes of 440 bp or 1200 bp were obtained (Figure 2D). To examine whether the eGFP gene inserted into the genome affected viral morphology, virions of the rCVS-11-eGFP strain were observed using electron microscopy (Figure 2E). Six randomly selected virions of the rCVS-11 and rCVS-11-eGFP strains were measured. The mean diameters of the rCVS-11-eGFP and rCVS-11 virions were 81.1 ± 13.3 and 80.1 ± 12.4 nm and the lengths 165.7 ± 18.7 nm and 163.4 ± 24.7 nm, respectively. The lengths and diameters of the two strains showed no significant differences (*p* > 0.05), and the inserted eGFP gene did not affect viral morphology. 

### 3.2. Growth Properties of rCVS-11-eGFP

**Figure 3:**
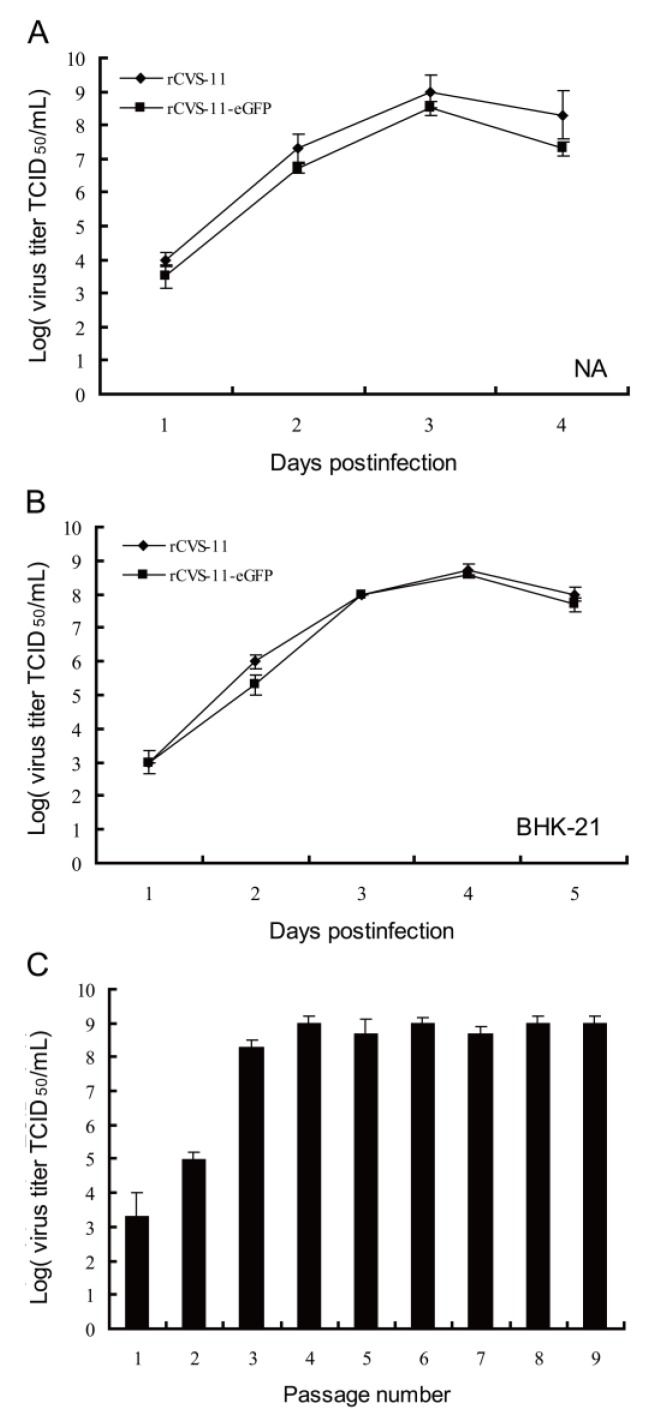
Growth characteristics of the rCVS-11 and rCVS-11-eGFP strains in NA and BHK-21 cells. (**A**, **B**) NA and BHK-21 cells were infected with rCVS-11 and rCVS-11-eGFP at an MOI of 0.1, and the viruses were titrated at different day p.i. The results are the means ± standard error of the mean (SEM) from titers in triplicate per day p.i. (*p* > 0.05); (**C**) Viral serial passage in BHK-21 cells. BHK-21 cells were infected with the rCVS-11-eGFP strain at an MOI of 0.1, and the supernatants from each passage were harvested at 96 h p.i.; the viral titers were determined in triplicate (*p* > 0.05). The values shown are the means ± SEM.

To determine whether an inserted eGFP gene in the rCVS-11 genome affected viral replication, NA, BHK-21, and 293T cells were infected in triplicate with the rCVS-11 or rCVS-11-eGFP strain at an MOI of 0.1. At 24 h, 48 h, 72 h, 96 h, and 120 h p.i., the supernatants of the infected cells were collected for virus titration. The growth curves of rCVS-11-eGFP were similar to rCVS-11 in these cell lines (*p* > 0.05; Figure 3A,B). rCVS-11-eGFP peaked at 1 × 10^8.3^ and 1 × 10^7.7^ TCID_50_/mL in the NA and BHK-21 cell lines, respectively.

To ascertain whether the rCVS-11-eGFP strain was genetically stable and increased the virus yield by adapting to cultured cells, we serially passaged the rCVS-11-eGFP strain in the BHK-21, 293T, and NA cell lines and found that the rCVS-11-eGFP titers in the supernatant gradually peaked at 10^8.3^ TCID_50_/mL at the 10th passage (Figure 3C), with stable expression of eGFP up to at least 20 passages (data not shown). The pathogenicity results showed that the 10^4^ TCID_50_ rCVS-11-eGFP and wtCVS-11 strains were 100% fatal to three-day-old suckling mice by the i.c. route. Although only 80% of the six-week-old mice developed clinical signs and died, the wtCVS-11 strain caused 100% mortality (data not shown).

### 3.3. Comparative Analysis of VNA Titers Determined by Standard FAVN and FAVN-eGFP Methods

**Figure 4:**
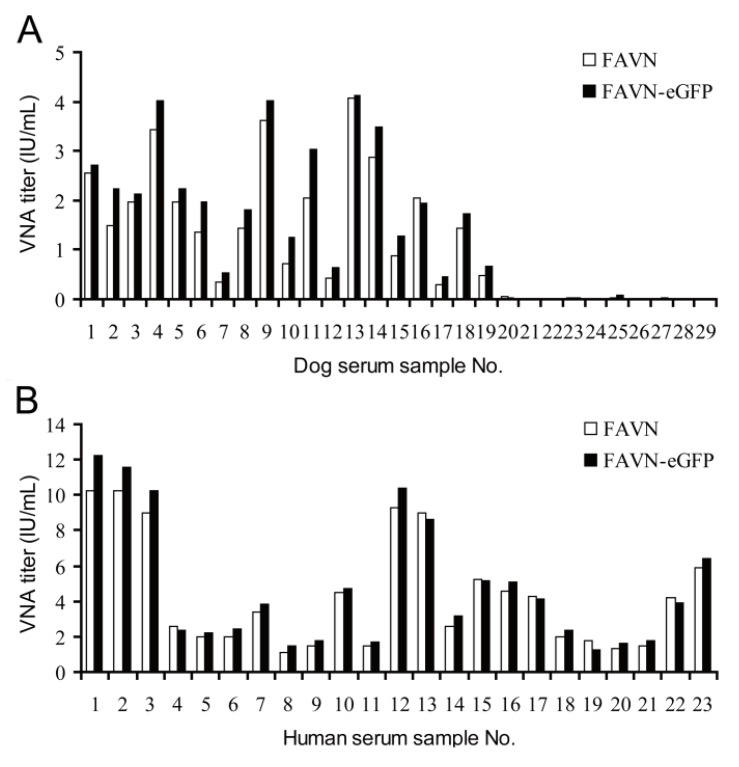
Comparison of rabies VNA titers determined with rCVS-11-eGFP and wtCVS-11. (**A**) Nineteen positive serum samples from vaccinated dogs (No. 1–19) and ten negative dog serum samples (No. 20–29) were assayed for rabies VNA titers using wtCVS-11 and rCVS-11-eGFP; (**B**) Twenty-three positive human serum samples from volunteers (No. 1–23) were assayed in the same manner as above. The results obtained with the two viruses were compared using a paired t-test, and no significant difference was observed.

To evaluate whether the novel FAVN-eGFP method was specific and accurate for the titration of virus-neutralizing antibody in serum samples, the VNA titers of the same serum were measured by two methods: (1) standard FAVN using the CVS-11 strain as a challenge virus and detection with an FITC-conjugated anti-N protein monoclonal antibody, the results from the OIE Reference Laboratory of rabies diagnosis; (2) the FAVN-eGFP method using rCVS-11-eGFP as a challenge virus and detection with eGFP expression without fluorescein staining under a fluorescent microscope. A total of 42 RABV-positive serum samples, including 19 from dogs (Figure 4A) and 23 from human volunteers (Figure 4B) were titrated with the two methods. As shown in Figure 4A and B, the FAVN-eGFP results showed slightly higher titers than the standard FAVN; however, there were no significant differences (*p* > 0.05, paired t-test). The titration of 10 negative canine sera (Figure 4A No. 20–29) with the two virus strains showed the same results (*p* > 0.05). The results obtained from samples from non-vaccinated dogs by both methods were in agreement. A comparison of the titers from vaccinated dogs and humans showed 96.6% agreement. However, the agreement was better when detection of the canine serum samples than that of the human serum samples. The statistical analyses of reproducibility obtained from FAVN-eGFP method showed no significant difference (data not shown). In addition, the eGFP expressed was still visible after fixation with 4% paraformaldehyde and preservation at 4 °C for a few months (data not shown). 

## 4. Discussion

Human rabies cases in developing countries have continued to increase in recent years. In a national epidemiological survey, 95% of patients had been bitten by dogs, 3% had been exposed to cats, and only 2% had been infected by other animals [4]. Therefore, it is important to control and prevent rabies in dogs in order to eliminate human rabies in China and other developing countries. 

The most widely used methods for the determination of RABV neutralizing antibody levels are the mouse neutralization test (MNT), RFFIT, and FAVN. MNT is time-consuming and costly; RFFIT has several advantages over MNT, such as reduced manpower and allowing for many samples to be tested at one time, though may result in subjective differences; the result readings of RIFFT necessitate the manual counting of fluorescent foci and therefore different results may result from different readers. The FAVN test is a modified method or an adjusted RFFIT, which belongs among the standard WHO methods for the determination of antibody titers. RFFIT requires the manual counting of the number of fluorescent foci in virus-infected cells [11], whereas FAVN is objective and suitable for large-scale samples, thereby enabling automated high-throughput detection. The conventional FAVN and RFFIT methods require a FITC-conjugated monoclonal antibody to identify positive or negative cells by the green fluorescence of FITC using fluorescence microscopy. However, good-quality FITC-conjugated anti-N protein monoclonal antibodies are scarce in developing countries. Additionally, the FITC-conjugated anti-N protein monoclonal antibody requires a good transport environment or is likely to be degraded during long-distant transport.

Although the introduction of an eGFP gene to the vaccine strains ERA, Hep-Flury and CTN-181 are safer than CVS-11, it is better to evaluate the effectiveness of vaccines with a pathogenic challenge virus such as CVS-11 strain than a vaccine strain itself. The CVS-11 strain has been widely approved as a challenge virus in RABV neutralizing antibody tests [13]. The eGFP was accumulated and easily observed in infected cells by fluorescence microscopy. In this study, an rCVS-11-eGFP recombinant virus was generated based on the reverse genetics of the laboratory-fixed CVS-11 strain and was applied to rapid virus-neutralizing antibody test termed FAVN-eGFP. The identifications of DFA, RT-PCR, and detection of eGFP expression showed that the recombinant virus rCVS-11-eGFP strain was successfully rescued and the rCVS-11-eGFP was typical bullet-shaped. The growth curves showed that the inserted eGFP gene did not affect the replication of the recombinant virus rCVS-11-eGFP. In addition, eGFP was stably expressed in rCVS-11-eGFP-infected BHK-21 (Figure 3C) and 293T cells (data not shown). This is consistent with other reports that the eGFP gene inserted in the genome of ERA [14], Hep-Flury [24], and CTN-181 [16] vaccine strains did not affect viral replication. The pathogenicity had no significant differences between the rCVS-11-eGFP and wtCVS-11 strains in mice (data not shown). The rCVS-11-eGFP used as a detective antigen was applied to determine the levels of neutralization antibody from 52 serum samples. The results showed good agreement with the results from the OIE Reference Laboratory of rabies diagnosis with the FAVN test. In addition, our data showed that there were no significant differences in the sensitivity and specificity between FAVN and FAVN-eGFP. The two methods were compared using 42 positive and 10 negative serum samples, and the results are expressed in IU/mL. The results obtained from samples from non-vaccinated dogs by both methods were in agreement. A comparison of the titers from vaccinated dogs and humans showed 96.6% agreement. However, the agreement was better with detection of the canine serum samples than that of the human serum samples. These results suggested that the FAVN-eGFP was more suitable for detection of animal serum samples. With regard to the accuracy of the results, the preparation and performance of the tests, and their respective costs, FAVN-eGFP appears to be better than FAVN. FAVN-eGFP does not require consecutive fixation and staining. Thus, FAVN-eGFP provides an alternative to FITC-conjugated monoclonal antibodies. The sera with titers close to 0.5 IU/mL were classified as positive by FAVN-eGFP and negative by FAVN, which could be explained by the different detection sensitivity between FAVN-eGFP using the visualization of accumulated expressed eGFP and FAVN using antigen-antibody binding. However, there were no significant differences in the sensitivity between the two methods. Animals and humans should be carefully treated when their VNA values are close to 0.5 IU/mL [21]. Thus, adapting FAVN-eGFP method as a routine VNA assay, whether the VNA titers approximately 0.5 IU/mL is enough protective have to be carefully decided in consideration for the titers obtained from FAVN-eGFP is higher than those from standard FAVN. These findings are consistent with previous reports that the RFFIT-GFP method using the recombinant rHep-Flury-GFP virus as a challenge virus is a useful and reliable tool for measuring VNA titers [24]. Samples whose titers are around 0.5 IU/mL were determined several times with FAVN-eGFP. Statistical analyses showed that there are no significant differences of the titers on the same serum samples. This is a profile of reproducibility. Therefore, when the titers are around 0.5 IU/mL by the FAVN-eGFP method, we should confirm with conventional FAVN and RFFIT. The differences between the RFFIT-GFP and RFFIT might be due to different antigenic conformation in G proteins between the challenge viruses strains used in the VNA assay. However, FAVN-eGFP uses the same CVS-11 strain as a challenge virus as the FAVN test. There is also the potential to mechanically scan the wells of 96-well plates using a fluorescence plate reader to automate the scoring of VNT assays, which would allow for high-throughput sample detection. 

## 5. Conclusions

A recombinant rabies virus rCVS-11-eGFP expressing eGFP is generated based on an infectious clone of wtCVS-11 strain. The eGFP gene doesn’t affect growth property and pathogenicity of rCVS-11. The rCVS-11-eGFP is used as a detection antigen to determine the levels of rabies VNAs in 23 human and 29 canine sera, and this technique is called the FAVN-eGFP method. The values obtained from FAVN-eGFP show 96.6% agreement with the results from traditional FAVN test. The FAVN-eGFP method allows rapid, economical, specific, and high-throughput assessment for the titration of rabies VNAs. 
